# Microgravity effects on nonequilibrium melt processing of neodymium titanate: thermophysical properties, atomic structure, glass formation and crystallization

**DOI:** 10.1038/s41526-024-00371-x

**Published:** 2024-03-06

**Authors:** Stephen K. Wilke, Abdulrahman Al-Rubkhi, Chihiro Koyama, Takehiko Ishikawa, Hirohisa Oda, Brian Topper, Elizabeth M. Tsekrekas, Doris Möncke, Oliver L. G. Alderman, Vrishank Menon, Jared Rafferty, Emma Clark, Alan L. Kastengren, Chris J. Benmore, Jan Ilavsky, Jörg Neuefeind, Shinji Kohara, Michael SanSoucie, Brandon Phillips, Richard Weber

**Affiliations:** 1 Materials Development, Inc., Evanston, IL 60202 USA; 2 X-ray Science Division, Advanced Photon Source, Argonne National Laboratory, Lemont, IL 60439 USA; 3 Japan Aerospace Exploration Agency, Tsukuba, Japan; 4 Center for High Technology Materials, University of New Mexico, Albuquerque, NM 87106 USA; 5 Inamori School of Engineering at the New York State College of Ceramics, Alfred University, Alfred, NY 14802 USA; 6 ISIS Neutron & Muon Source, Rutherford Appleton Laboratory, Chilton, Didcot, Oxon, OX11 0QX, UK; 7 Neutron Science Division, Spallation Neutron Source, Oak Ridge National Laboratory, Oak Ridge, TN 37831 USA; 8 National Institute for Materials Science, Tsukuba, Japan; 9 NASA Marshall Space Flight Center, Huntsville, AL 35812 USA

**Keywords:** Structure of solids and liquids, Techniques and instrumentation, Structure of solids and liquids

## Abstract

The relationships between materials processing and structure can vary between terrestrial and reduced gravity environments. As one case study, we compare the nonequilibrium melt processing of a rare-earth titanate, nominally 83TiO_2_-17Nd_2_O_3_, and the structure of its glassy and crystalline products. Density and thermal expansion for the liquid, supercooled liquid, and glass are measured over 300–1850 °C using the Electrostatic Levitation Furnace (ELF) in microgravity, and two replicate density measurements were reproducible to within 0.4%. Cooling rates in ELF are 40–110 °C s^−1^ lower than those in a terrestrial aerodynamic levitator due to the absence of forced convection. X-ray/neutron total scattering and Raman spectroscopy indicate that glasses processed on Earth and in microgravity exhibit similar atomic structures, with only subtle differences that are consistent with compositional variations of ~2 mol. % Nd_2_O_3_. The glass atomic network contains a mixture of corner- and edge-sharing Ti-O polyhedra, and the fraction of edge-sharing arrangements decreases with increasing Nd_2_O_3_ content. X-ray tomography and electron microscopy of crystalline products reveal substantial differences in microstructure, grain size, and crystalline phases, which arise from differences in the melt processes.

## Introduction

Manufacturing processes suitable to reduced gravity and microgravity environments must be developed to advance the low Earth orbit (LEO) economy, utilize resources from locations beyond Earth, and actualize self-sustainable extraterrestrial habitats with closed-loop materials recycling^1^. Melt processing is one common manufacturing step with particularly challenging differences between terrestrial and microgravity conditions. For microgravity, Earth-based melt processes must be adapted to the lack of buoyancy-driven convection, density-induced sedimentation, and gravitational forces to direct fluid flow.

Additive manufacturing techniques like selective laser melting are some examples of melt processing that may be suitable for reduced gravity environments^1–4^. In such processing techniques, the product’s shape, chemical phase, and material microstructure are all influenced by the interplay of energy deposition and the melt’s thermophysical properties, such as viscosity and surface tension^5–7^. These processing-structure-property relationships illustrate that accurate measurements of liquid thermophysical properties are crucial to enabling space-based manufacturing. Additionally, melt processing often concludes with solidification from nonequilibrium states, such as supercooled liquids. Because fluid flow and heat transfer differ between Earth and microgravity environments, different states of nonequilibrium will likely be encountered that provide new challenges and new opportunities for materials discovery and manufacturing technology. Such opportunities may manifest through leveraging of curated large datasets for low-level machine learning and algorithm development, also known as Digital Twin interests. This would drive performance and design improvements that would have both terrestrial and space-based benefits in the form of newer materials, processes, and efficiencies.

Historically, microgravity research on melt processing and liquid thermophysical properties has focused on metallic materials, which have been studied in the TEMPUS^8,9^ campaign and the Electromagnetic Levitator Facility^10^ onboard the International Space Station (ISS). Until recently, less attention has been given to ceramic and glass materials. One notable exception is ZBLAN glass (ZrF_4_-BaF_2_-LaF_3_-AlF_3_-NaF). ZBLAN is a candidate material for improved, low-loss optical fiber communications because it has a theoretical optical attenuation one to two orders of magnitude lower than that of silica^11^. However, ZBLAN is difficult to process due to its sensitivity to water and small working temperature range, i.e. the range between its glass transition and crystallization point. Early microgravity experiments on ZBLAN suggested that crystallization may be suppressed in reduced gravity^12^, thereby widening the working temperature range and hypothetically making it possible to manufacture higher-quality ZBLAN fiber. The increase in crystallization temperature was speculated to arise from a lack of buoyancy-driven convection^13^, though a full mechanistic explanation remains lacking. Nonetheless, multiple commercial efforts are currently pursuing space-based processing of ZBLAN fibers^14,15^.

Microgravity research on oxide ceramics, glasses, and their manufacturing remains in its infancy. In 2016, the Japanese Aerospace Exploration Agency commissioned the Electrostatic Levitation Furnace (ELF)^16^ onboard the International Space Station, which is well suited for high-temperature melt processing of oxides. The ELF uses three orthogonal pairs of electrostatic transducers to position and levitate spheroidal samples ~2 mm in diameter. During levitation, samples can be heated with four 980 nm lasers, achieving melts up to ~3070 °C^17^. Silhouette imaging and optical pyrometry enable volume measurements of molten samples as a function of temperature, from which the sample density and thermal expansion coefficient can be calculated based on its preflight and/or postflight mass^18^. Liquid viscosity and surface tension can also be measured using a droplet oscillation technique^19^. By applying a sinusoidal variation to the transducers’ voltages along one axis, oscillations in the liquid can be induced. If the excitation frequency matches the natural resonance of the droplet, analytical expressions can relate the resonance frequency and characteristic time for oscillation damping (upon stopping the excitation) to the liquid’s surface tension and viscosity, respectively^20^. These relationships depend on assumptions of fluid quiescence, which is why microgravity provides a unique advantage for measuring these thermophysical properties at high temperatures. The ELF can operate at up to 2 bar of gas pressure and can utilize oxygen-containing gases, which are desirable for oxide studies.

Using the ELF, we are reporting a comprehensive comparison of melt processing on Earth versus in microgravity for one particular oxide, a neodymium titanate (NT) with nominal composition 83TiO_2_–17Nd_2_O_3_. The NT composition is of interest technologically as an optical material: its glass has a high refractive index (*n* > 2.1)^21,22^ and a wide transmission window spanning the visible and infrared range out to ~5 μm^21,23^. NT is also of fundamental scientific interest as a fragile liquid^24^ and poor glass-former^25^, with an atomic network of octahedrally coordinated Ti-O polyhedra^26^ atypical of oxide glasses. Here, we compare the cooling rates, glass formation, and recalescence of molten and supercooled NT processed in the ELF and in a terrestrial aerodynamic levitator. Density is reported as a function of temperature for the equilibrium liquid (ca. 1470–1850 °C), supercooled liquid (ca. 780–1470 °C), and glass (300–780 °C). The glass atomic structure is assessed with X-ray and neutron total scattering and Raman spectroscopy. Microstructures in both glassy and crystalline samples are probed with X-ray microtomography, X-ray small-angle scattering, and electron microscopy. This suite of characterizations aims to benchmark the microgravity melt processing technique against established terrestrial methods, while identifying the differences and advantages offered by space-based melt processing.

## Results

### Melt processing and glass formation

Samples of 83TiO_2_–17Nd_2_O_3_ nominal composition (83 mol. % TiO_2_; 17 mol. % Nd_2_O_3_) were prepared as spheroids ~2 mm in diameter (20–23 mg), and these samples were heated, melted, and quenched using two different containerless processing techniques. In microgravity, samples were levitated in the ELF^16^ using electrostatic forces and heated with four 980 nm lasers positioned around the sample like corners of a tetrahedron, with the sample in the center. On Earth, samples were processed in an aerodynamic levitator using a gas stream passing through a converging-diverging nozzle, and the top of the sample was heated with a 10.6 μm CO_2_ laser^27^. In both techniques, the samples were held isothermally near 1850 °C, fully molten (*T*_m_ = 1467 °C^28^), for at least 30 s before turning off the heating lasers. The samples then cooled freely and, depending on the cooling rate, either vitrified into glass or crystallized from a supercooled state. The containerless processing techniques provide two major benefits for melt processing research: (i) avoiding chemical contamination from sample-crucible reactions at high temperatures, and (ii) removing any source of heterogeneous nucleation, so that supercooling and vitrification are easier at modest cooling rates (i.e., <10^3^ °C s^−1^) than in the presence of a container. Electrostatic levitation has been applied to liquids with a wide range of surface tensions, from propylene carbonate^29^ (43 mN m^−1^) to tungsten^30^ (~2500 mN m^−1^).

**Fig. 1:**
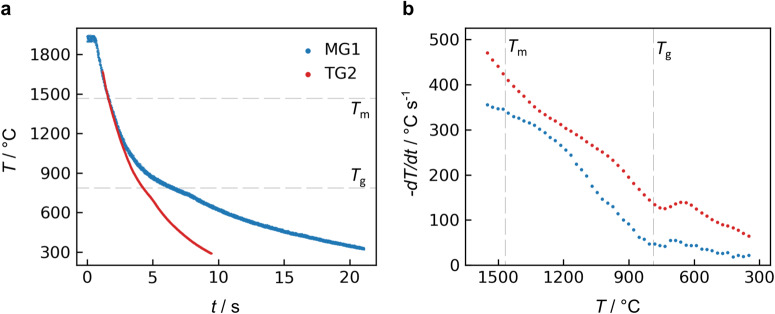
Cooling of melt quenched neodymium titanate (NT) in air. **a** Sample temperature vs. time after heating lasers were turned off, for melt processing in microgravity (MG1) and terrestrially (TG2). All temperatures are corrected for sample emissivity and window reflections. Both samples were cooled from ca. 1850 °C. **b** Cooling rate as a function of temperature.

Exemplary cooling curves for samples in microgravity (“MG1”) and on Earth (“TG2”) are compared in Fig. 1a. Both measurements used optical pyrometers with λ ~ 1.5 μm, but the range of the terrestrial pyrometer was limited to a maximum of 1650 °C. (Terrestrial sample temperature above 1650 °C was monitored with a pyrometer of different wavelength). Temperature measurement uncertainty is ca. ±30 °C for ELF and ±27 °C in the aerodynamic levitator (see Supplementary Discussion). The terrestrial and microgravity samples cooled from ~1850 to 300 °C in ~8.9 and 20.6 seconds, respectively. Figure 1b shows the corresponding cooling rates as a function of temperature. Comparing the two processing environments provides insight into the relative contributions of different heat transfer modes. At high temperatures, radiative heat transfer (~*T*^4^) is dominant, while at the lowest temperatures, the samples cool due to convection and/or conduction. In microgravity, buoyancy-driven convection is minimal, so the cooling rate of 20–60 °C s^−1^ observed below 800 °C is likely due to heat conduction through the gas surrounding the sample. For any given temperature, the terrestrial cooling rate is 40–110 °C s^−1^ larger than in microgravity. This difference in cooling rate represents the contribution from forced convective cooling by the levitation gas around the sample.

Both the terrestrial and microgravity cooling rates in Fig. 1b exhibit slope fluctuations at several temperatures. The most reproducible fluctuation is a transient increase in cooling rate ca. 570–740 °C, which was always observed in repeated terrestrial measurements and occurs just below the glass transition at *T*_g_ = 786 °C^21^. This fluctuation is most likely an artifact caused by a combination of the pyrometer measurement and NT optical properties. NT glasses exhibit several absorption bands in the visible and near-infrared range due to Nd^3+^ optical activity^21^; the sample transparency and fluorescence near the pyrometer wavelength may change with temperature. Two auxiliary tests in the aerodynamic levitator were conducted to test this hypothesis. First, NT cooling rates were measured with three different pyrometer wavelengths—λ = 0.9, 1.5, and 5.0 μm (Supplementary Fig. 1a, b)—and the magnitude of the cooling rate fluctuation varied significantly, appearing most strongly for λ = 0.9. Second, cooling curves for an optically inactive lanthanum titanate sample (83TiO_2_-17La_2_O_3_) did not exhibit this cooling rate fluctuation (Supplementary Fig. 1c, d). These observations support the explanation that NT’s apparent fluctuation ca. 570–740 °C is a measurement artifact. In Fig. 1b, the cooling rate also exhibits more subtle slope fluctuations at other temperatures, and these may be due to slow sample rotation observed in both levitation techniques. The sample surface is known to have spatial temperature variations (e.g., ~30 °C for liquids at 1500 °C in the aerodynamic levitator^31^), so sample rotation could result in apparent temperature fluctuations where the pyrometer is focused.

In addition to the glass samples obtained by the cooling trajectories in Fig. 1, crystalline samples were also produced by melt quenching. Crystallization typically occurred from the supercooled liquid at temperatures of 900–1150 °C, evidenced by a visual recalescence and a momentary increase in the sample temperature of tens to hundreds of °C. One sample processed in microgravity was later found to contain trace amounts of crystals throughout the material (i.e., a glass-ceramic), despite its cooling curve not exhibiting recalescence.

### Density

**Fig. 2:**
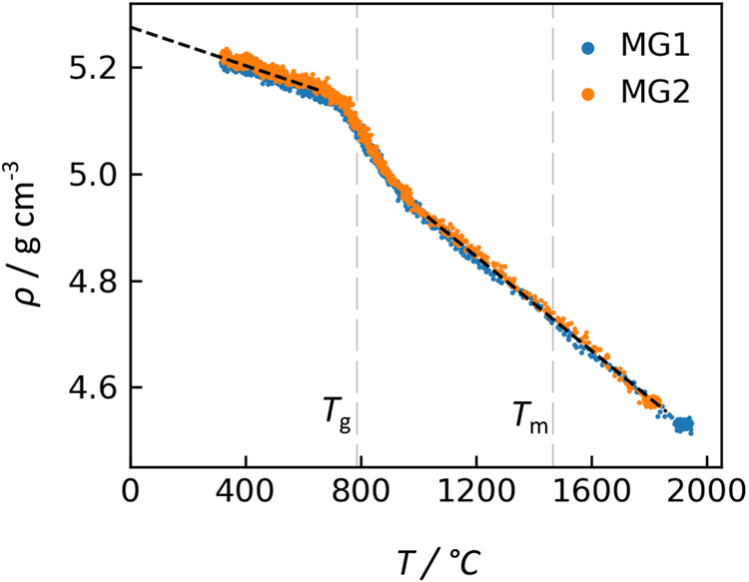
Density during melt quenching. Measurements of two replicate samples (MG1 and MG2) that formed glass during melt cooling in ELF. All temperatures are corrected for sample emissivity and window reflections, and density has been corrected for sample mass loss and internal porosity, as discussed in the main text. Linear fits to the glass and liquid thermal expansion are shown with dashed black lines.

The densities of two NT samples melt quenched and vitrified in microgravity are shown in Fig. 2. These density values were calculated using the sample mass after melt processing (i.e., after the sample return to Earth) and the volume deduced from silhouette backlight imaging of the levitated samples during cooling^18^. Samples’ post-processing masses were 0.3–1.0% lower than their initial masses, likely due to volatilization. X-ray tomography of the recovered samples revealed internal porosity of up to 0.25%, so the sample volumes were corrected for this porosity before calculating the density shown in Fig. 2. This correction is based on porosity observed in the glassy samples, so it is possible that the internal pore sizes were different in the molten state when volume measurements were collected.

The density vs. temperature curves in Fig. 2 agree within 0.4% for the replicate samples MG1 and MG2. Liquid density increases from 4.54 to 4.92 g cm^−3^ during cooling from 1860 to 1030 °C, and the trend with temperature is described well by a linear fit:

1 $$\rho \left(T\right)=5.37\left(1\right)-4.40(5)\times {10}^{-4}\times T{\rm{;MG}}1,1030\, < \,T\, < \,1860$$

2 $$\rho \left(T\right)=5.38\left(1\right)-4.43(6)\times {10}^{-4}\times T{\rm{;MG}}2,1030\, < \,T\, < \,1740$$

In these equations, *ρ* is in units of g cm^−3^, *T* is in units of °C, and the values in parentheses indicate a 95% confidence interval. The mean values of the linear equations for MG1 and MG2 are shown in Fig. 2 with a black dashed line. The slope value of −*dρ*⁄*dT* ~ 4.4 × 10^−4 ^g cm^−3^ °C^−1^ for the melt is in reasonable agreement with previously reported values for other titanate melts^32,33^: BaTiO_3_ with 3.4 × 10^−4^, and BaTi_2_O_5_ with 4 × 10^−4^.

For the glass, density increases from 5.15 to 5.22 during cooling from 670 to 300 °C, with a trend well described by a linear fit. However, this result should be accepted with some caution, since glass transparency at the pyrometer wavelength introduces temperature uncertainties that are difficult to quantify.

3 $$\rho \left(T\right)=5.27\left(1\right)-1.86\left(5\right)\times {10}^{-4}\times T{\rm{;MG}}1,300\, < \,T\, < \,670$$

4 $$\rho \left(T\right)=5.28\left(1\right)-1.76(4)\times {10}^{-4}\times T{\rm{;MG}}2,300 \,<\, T\, <\, 670$$

It is emphasized that the melt and supercooled liquid measurements are the most valuable result that the ELF instrument enables; glass density and its temperature dependence can be more easily and accurately measured by other ground-based techniques.

Extrapolation of the glass density to room temperature yields a value of 5.28 g cm^−3^, which is larger than expected from prior measurements^22^ (5.13 g cm^−3^, see Supplementary Discussion) and the compositionally similar crystalline phase (5.18 g cm^−3^ for Nd_4_Ti_9_O_24_^34^). However, this discrepancy of 2.9% is close to the relative uncertainty of 2.5% previously reported for ELF^19^.

More puzzling is the observation ca. 720–930 °C of a region with larger thermal expansion than the glass or the liquid. The steeper slope in Fig. 2 for this region generally would suggest some heat release by the sample, possibly by crystallization, but synchrotron X-ray scattering showed no sign of crystallinity. The process of melt quenching and vitrification is expected to show a density-temperature plot with two linear regions, one each for glass and liquid, so this third anomalous region encompassing *T*_g_ represents either a measurement artifact or a new, unexpected behavior. Several hypotheses for measurement artifacts were explored (see Supplementary Discussion). One explanation involves the sample becoming partially transparent at the pyrometer wavelength during cooling. If the transition to partial transparency began near 930 °C, then the pyrometer would start seeing some of the radiation emitted by the sample’s hotter interior. This would result in a higher reading than the surface temperature, until the sample interior had also cooled enough to become partially transparent. This could explain the steeper slope in Fig. 2 between 720 and 930 °C, yet it is not a fully convincing argument. If instead the data are accurate and this anomalous region is not due to a measurement artifact, the unexpected density-temperature relationship could be caused by some kind of liquid-liquid phase transition or other thermodynamic anomaly in this fragile liquid approaching *T*_g_^35^.

Density measurements are challenging for fragile liquids and their glasses because melt processing typically requires containerless conditions with small sample volumes. Glass density is typically measured using pycnometry of samples 2–3 mm in diameter, as was done by Arai et al.^22^ on lanthanum titanate glasses, though this technique can have significant measurement uncertainty for small sample sizes^26^. Melt densities have been measured using backlight imaging techniques in aerodynamic levitation, but partial occlusion of the sample and asphericity introduce an uncertainty of ~5%^33^. Improved precision with backlight imaging is possible using combined aerodynamic and acoustic levitation techniques^36^, which provide optical access to the entire sample. Given this context, electrostatic levitation and microgravity processing are useful tools for improving the precision of density measurements on fragile liquids.

### Glass atomic structure

The atomic structures in terrestrial and microgravity glass samples were probed using high-energy X-ray diffraction, neutron diffraction, and Raman spectroscopy. Although gravity is not expected to directly influence atomic arrangements, these characterizations remain important to benchmark the similarities and differences between terrestrial and microgravity processing.

We recently reported a thorough structural analysis of terrestrial NT glasses^26^, based on X-ray/neutron diffraction and structural modeling, so only brief comments are provided here on the interpretation of the scattering data, focusing specifically on differences between microgravity and terrestrial samples. The glass atomic network has been shown to comprise TiO_5_ and TiO_6_ polyhedra connected primarily via corner-sharing arrangements, with ~23% edge-sharing, while the Nd^3+^ ions act similarly to network modifiers^26^.

**Fig. 3:**
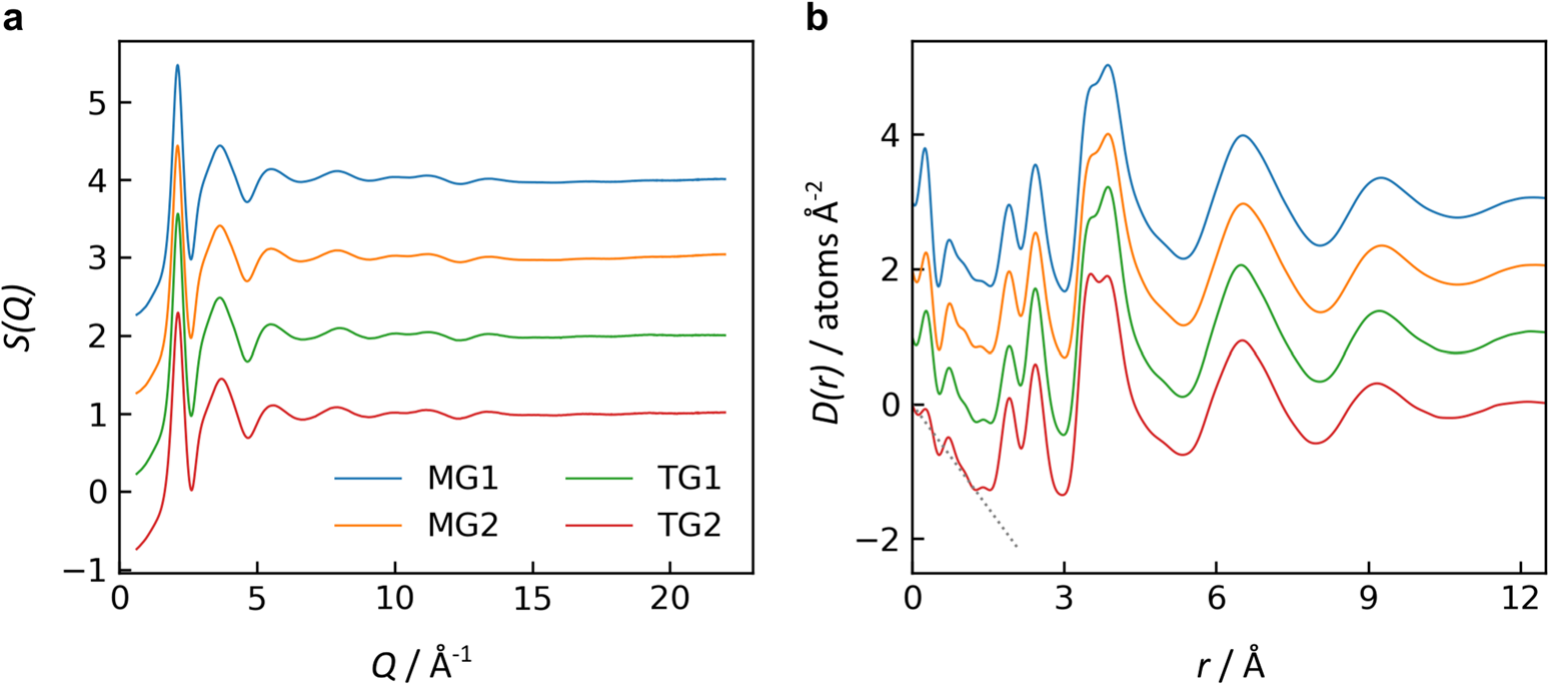
X-ray diffraction of NT glasses. **a** X-ray total structure factors and **b** differential pair distribution functions for two replicate glasses melt quenched in microgravity (MG1 and MG2) and two glasses melt quenched on Earth (TG1 with 20.5 mol. % Nd_2_O_3_; TG2 with 17.1 mol. % Nd_2_O_3_). The gray dotted line indicates the low-*r* slope in *D*(*r*) given by −4πρ, where ρ is the atomic number density. Curves are offset vertically for visual clarity.

X-ray total structure factors, *S*(*Q*), and differential pair distribution functions (PDFs), *D*(*r*), are shown in Fig. 3a, b for two glasses processed in microgravity (MG1 and MG2) and two glasses processed on Earth (TG1 and TG2). In the structure factors, the principal peak near *Q* = 2.13 Å^−1^ represents the packing of Ti-O polyhedra in the glass network. In the PDFs, the low-*r* region matches the anticipated slope of −4πρ, where *ρ* = 0.08163 atoms Å^−3^ is the atomic number density (equivalent to 5.10 g cm^−3^ for NT with 18.2 mol. % Nd_2_O_3_). The first PDF peak corresponds to the Ti-O pair correlation, with a mean bond distance of *r*_*TiO*_ = 1.92 Å and mean coordination number of *n*_*TiO*_ = 5.2 based on peak fitting. The second PDF peak is the Nd-O correlation, with *r*_*NdO*_ = 2.45 Å and *n*_*NdO*_ = 8.4. These values are somewhat different than those from the published structural model^26^: *r*_*TiO*_ = 1.984(11) Å and *n*_*TiO*_ = 5.72(6); *r*_*NdO*_ = 2.598(22) Å and *n*_*NdO*_ = 7.70(26). These discrepancies are larger than the typical uncertainty from peak fitting, e.g. ±0.5 for coordination numbers, and likely arise from slight compositional differences as discussed below. Also, peak fitting here is based on a single diffraction measurement, compared to the published structural model that was refined based on six independent diffraction measurements, which resolves many of the issues related to overlapping features of the different atomic partial pair correlations.

All glasses exhibit nearly identical structures. From the peak fitting, all terrestrial and microgravity glasses in Fig. 3b have Ti-O and Nd-O mean bond distances within 0.01 Å and coordination numbers within 0.1 of one another. (These variations are smaller than the typical uncertainties for peak fitting.) There are only subtle differences between samples, most easily discerned in the third peak of the PDFs. This third peak corresponds to the cation-cation pair correlations, with overlapping contributions for Ti-Ti, Ti-Nd, and Nd-Nd. In Fig. 3b, PDFs are shown for terrestrial glasses TG1 and TG2, containing 20.5(2) or 17.1(2) mol. % Nd_2_O_3_, respectively. TG1 has greater asymmetry in the 3rd PDF peak, as compared to the nearly nominal composition in TG2, with stronger intensity on the higher-*r* shoulder where the Nd-Nd correlation is anticipated. This difference in the PDFs is expected, since compositional changes alter the weighting factors that determine how much each atomic partial pair contributes to the overall PDF^37^. Compositional enrichment by Nd would increase the weighting factor for Nd-containing atomic partial pair correlations. For the microgravity glasses MG1 and MG2, their 3rd PDF peaks are more similar to TG1 than TG2. For this reason, we hypothesize that the microgravity glasses are near ~19 mol. % Nd_2_O_3_. This discrepancy vs. the nominal composition (17 mol. % Nd_2_O_3_) can be explained by (i) the typical compositional variation of ~1 mol. % Nd_2_O_3_ observed between replicate beads, and (ii) the ~1% mass loss of samples processed in microgravity. (Compositional measurements were not performed on the microgravity glasses due to concerns about the required surface grinding and polishing for EDS.)

X-ray measurements were collected at 20 locations across each glass sample, and the structure factors were found to be consistent, indicating homogeneity throughout each sample.

Neutron diffraction is a technique for assessing atomic structure that is complementary to X-ray diffraction. Because X-rays scatter more strongly from heavier elements, the X-ray structure factor for NT is predominantly weighted by the atomic pair correlations that contain Nd or Ti. Neutron scattering is much more sensitive to O than X-rays are, so the neutron structure factor contains more information on the O local environments^26^. For this reason, neutron diffraction was also conducted as a probe for structural differences between the glasses processed in microgravity vs. terrestrially.

**Fig. 4:**
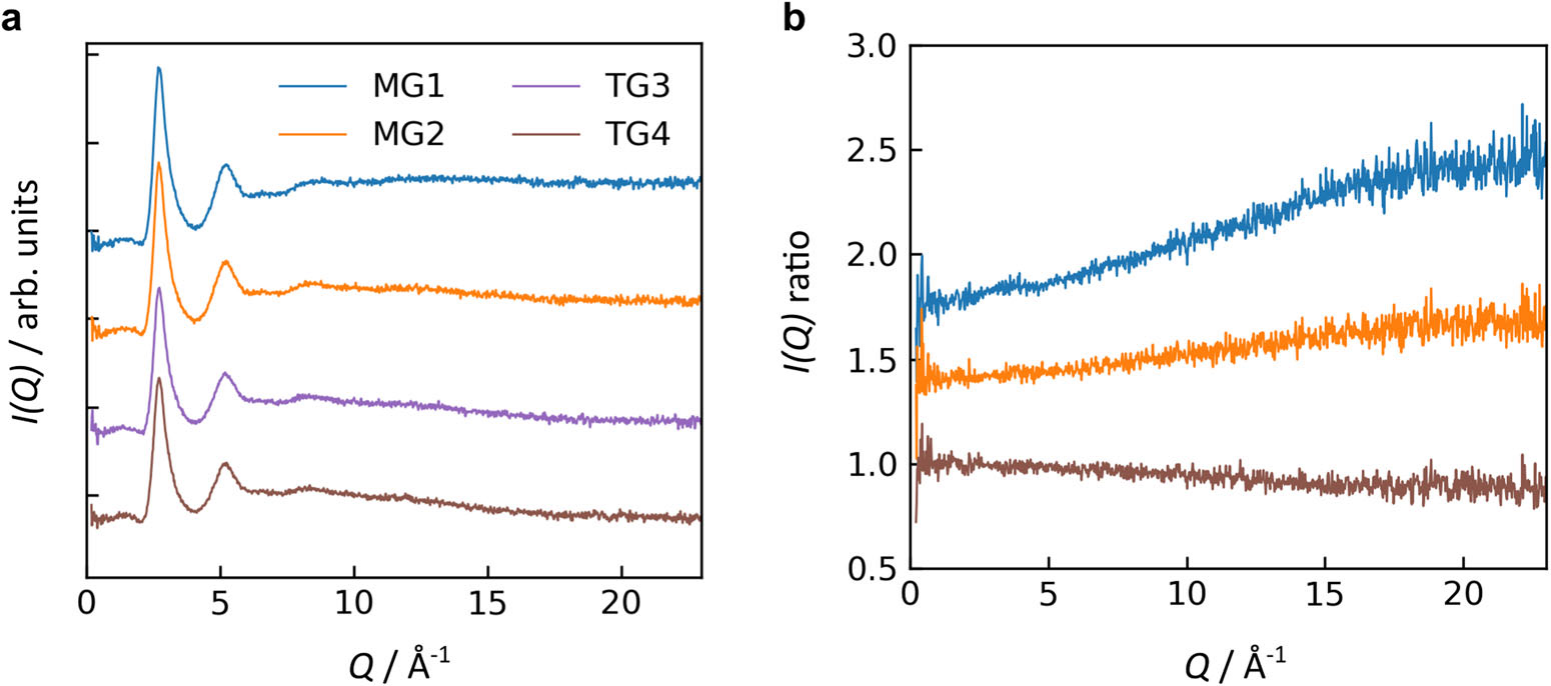
Neutron diffraction of NT glasses. **a** Scattering intensity after background subtraction and normalization to a vanadium standard; **b** the ratio of scattering intensity for each sample to that of one terrestrial glass. Data is shown for two replicate glasses melt quenched in microgravity (MG1 and MG2) and two glasses melt quenched on Earth (TG3 and TG4, both containing 17.6(2) mol. % Nd_2_O_3_). Curves are offset vertically for visual clarity.

Neutron diffraction measurements are shown in Fig. 4 for glasses melt processed in either microgravity (MG1 and MG2) or on Earth (TG3 and TG4). The scattering intensities, *I*(*Q*), are similar in Fig. 4a for all samples. To discern subtle differences, the scattering intensities in Fig. 4a were each divided by *I*(*Q*) for one of the terrestrial glasses (TG3), yielding the *I*(*Q*) ratios in Fig. 4b. The most notable differences between samples are their slopes in Fig. 4b, which are likely due to slight variations in samples’ positions relative to the neutron beam. Another possible explanation is differences in inelastic scattering, perhaps arising from hydrogen content in the samples. Optical measurements of rare-earth titanate glass have shown that they generally have low hydroxide impurity^38^, so it is likely that any hydrogen differences in neutron scattering arise from water. Otherwise, the *I*(*Q*) ratios are nearly featureless aside from some faint fluctuations near *Q* = 4 Å^−1^.

**Fig. 5:**
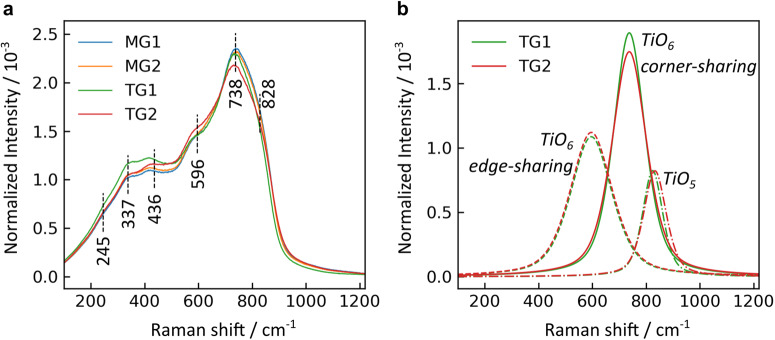
Raman spectra of NT glasses. **a** Unpolarized spectra for two glasses melt quenched in microgravity (MG1 and MG2), and two glasses melt quenched terrestrially (TG1 with 20.5 mol. % Nd_2_O_3_; TG2 with 17.1 mol. % Nd_2_O_3_). Annotated bands are discussed in the text. **b** Fitted component bands corresponding to stretching of Ti-O polyhedra that comprise the glass structural network, comparing compositional effects for TG1 and TG2.

The reduced and normalized Raman spectra for microgravity and terrestrial glasses are shown in Fig. 5a. Each Raman spectrum displays expected features of highly coordinated titanate polyhedra, as discussed by Su et al.^39^, and bears a strong resemblance to a spectrum recently reported for 83TiO_2_-17La_2_O_3_^38^. These features include a highly polarized envelope spanning roughly 550–900 cm^−1^, belonging to stretching modes of titanate polyhedra with coordination numbers larger than 4, and a depolarized envelope between 300–550 cm^−1^ containing bending modes.

For the two terrestrial glasses (TG1 and TG2 with 20.5(2) and 17.1(2) mol. % Nd_2_O_3_, respectively), the spectra were adequately reproduced using six peaks. The peak fitting results are plotted in Supplementary Fig. 2, and the band assignments are summarized in Supplementary Table 1 and described as follows. First, there is a weak and relatively broad band at 245 cm^−1^, which overlaps with the edge of the instrument range and, as such should not be over interpreted. Vibrational activity in this region is often characteristic of network deformation modes. Rare-earth cations can also display RE-O stretching activity in the region between 200–350 cm^−1^, which could contribute to the 245 cm^−1^ component. This RE-O activity likely contributes strongly to a band at 337 cm^−1^, which increases in intensity with increasing Nd_2_O_3_ content. For comparison, far infrared analysis of lithium rare-earth orthoborate glasses containing 20 mol. % RE_2_O_3_ displayed peak vibrational activity between 320–380 cm^−1^, depending on the RE^3+^ field strength^40^. Raman measurements on the same glasses^40^ as well as binary rare-earth borates displayed similar features^41^. Considering that the 337 cm^−1^ band displays a ~23% intensity increase as the Nd_2_O_3_ content increases just a few percent, it is likely that bending modes of some titanate arrangements are also contributing here. The band at 436 cm^−1^ is solely due to bending of Ti-O-Ti linkages, based on its depolarized nature (Supplementary Fig. 3) and higher frequency than is expected from RE-related activity.

The high-frequency envelope is best described by three component bands. The prominent 738 cm^−1^ is consistent with the stretching activity of corner-sharing TiO_6_ moieties^39^. The broadest peak, centered at 596 cm^−1^, is most likely due to edge-sharing TiO_6_ units and is just slightly below the 600–650 cm^−1^ range where the corresponding feature is found in anatase and rutile^39,42^. Finally, the peak at ~828 cm^−1^ is most likely due to TiO_5_ polyhedra. Activity in the 830–930 cm^−1^ range is generally unclear in TiO_2_-bearing glasses^43–45^: it has often been attributed to TiO_5_ species, as in fresnoite, but recently Santos et al. argued the assignment of an 825 cm^−1^ band as belonging to TiO_4_ species with nonbridging oxygen^43^, which are known to be at higher frequencies when all bridging^42,46,47^. Since these NT glasses are expected to have ~26% of Ti in TiO_5_ and only a small population of TiO_4_ (~1%)^26^, the band at ~828 cm^−1^ here is assigned to TiO_5_. During the fitting procedure, a band was positioned between 950–1050 cm^−1^ to simulate the symmetric stretch of possible TiO_4_, but the fitting optimization returned zero intensity for this component, consistent with the previously published structural model^26^.

The compositional variation between terrestrial glasses TG1 and TG2 is subtle but discernible. Mainly, as the Nd_2_O_3_ content increases (TG2 < TG1), the intensities of the 337 cm^−1^ and 738 cm^−1^ bands increase, and the intensity of the 596 cm^−1^ band decreases. A comparison of these fitted component bands for corner- and edge-sharing Ti-O polyhedra is shown in Fig. 5b. These differences may be due to an increase in corner-sharing TiO_6_ as the added Nd^3+^ cations depolymerize the Ti-O network and destroy edge-sharing arrangements. Since the corner-sharing motifs are already the majority species, such a scenario would lead to a less diverse structural landscape, and this may be the mechanism behind the decreased FWHM values fitted for the high-frequency stretching bands (Supplementary Table 1).

Raman spectra for the two glasses processed in microgravity (MG1 and MG2) are similar to each other, and their spectra are more similar to TG1 with 20.5(2) mol. % Nd_2_O_3_ than to TG2 with 17.1(2) mol. % Nd_2_O_3_. This observation is consistent with the compositional variation suggested by the X-ray diffraction data. The microgravity glasses and TG1 show some differences in the low-frequency range (i.e., 337 and 436 cm^−1^ bands).

### Microstructure of glasses and crystals

**Fig. 6:**
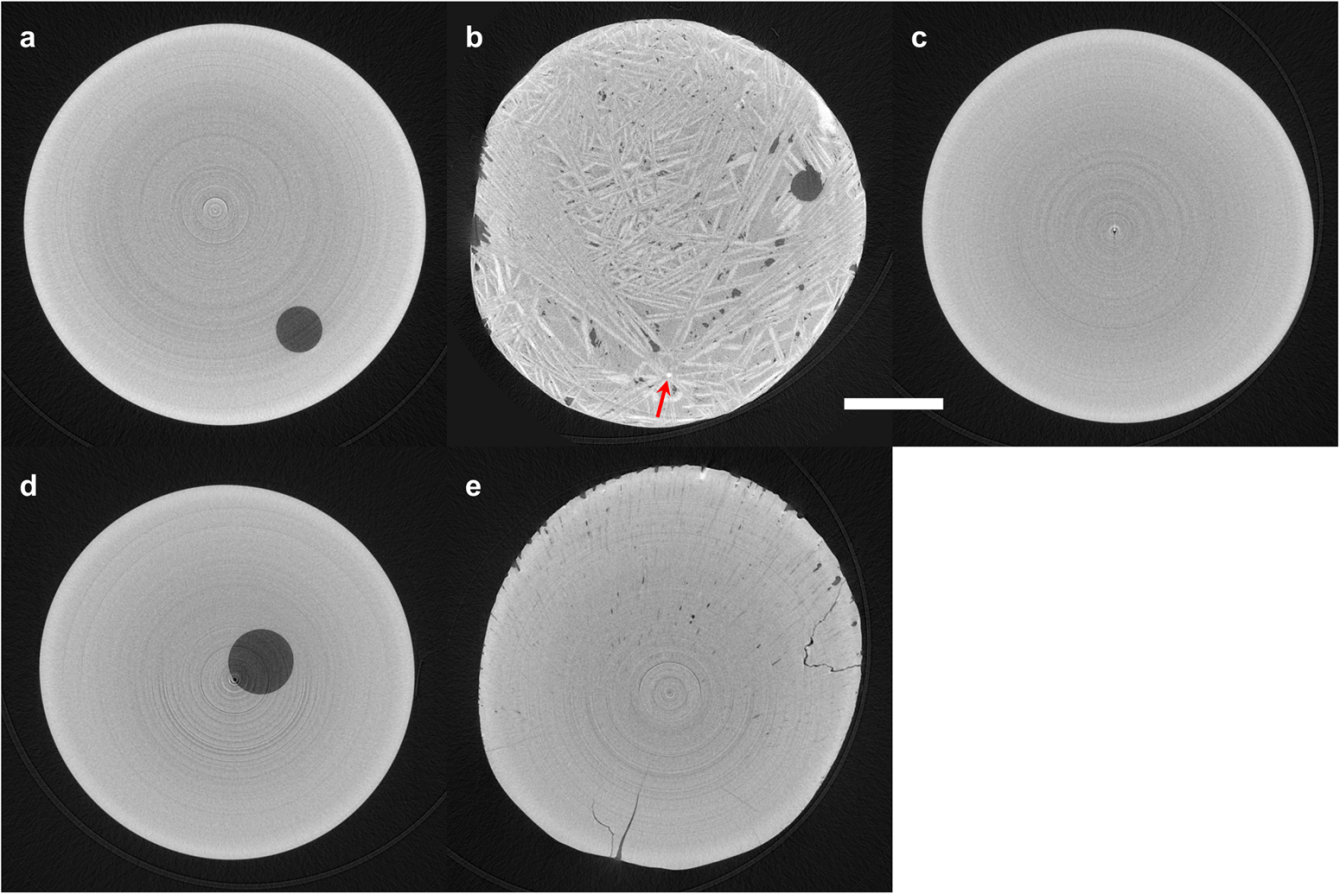
Cross-sections of NT samples from X-ray microtomography. **a** Glass, **b** crystal, and **c** glass-ceramic formed during melt processing in microgravity. **d** Glass and **e** crystal formed during terrestrial melt processing. Scale bar is 500 μm. The red arrow indicates the likely crystal nucleation point in **b**.

**Fig. 7:**
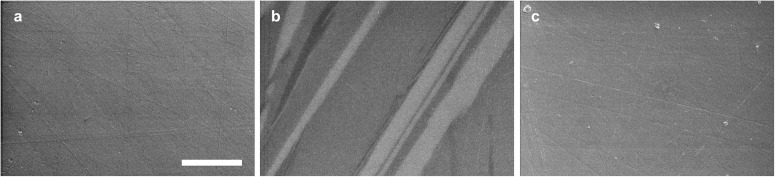
Scanning electron microscope images of NT samples. **a** Terrestrial glass; **b** microgravity crystal; **c** microgravity glass-ceramic. Scale bar is 3 μm.

Glass samples prepared in microgravity and terrestrially all appeared homogeneous in optical microscopy, X-ray tomography, and scanning electron microscopy (SEM). The three-dimensional reconstructions from tomography exhibited no density contrast, except for a few small internal voids. Figure 6 provides exemplary cross-sections from tomography, and video animations showing the three-dimensional reconstructions are in Supplementary Movies 1–5. The two microgravity glasses (Fig. 6a) each contained 2–3 pores ca. 0.24 mm in diameter or smaller, yielding total porosities of 0.10–0.25%. These pores were spherical, suggestive of gas bubbles present in the melt. The terrestrial glasses (Fig. 6d) similarly exhibited a few, small internal pores. SEM imaging of terrestrial glass cross-sections revealed no features except for surface scratches from polishing (Fig. 7a).

**Fig. 8:**
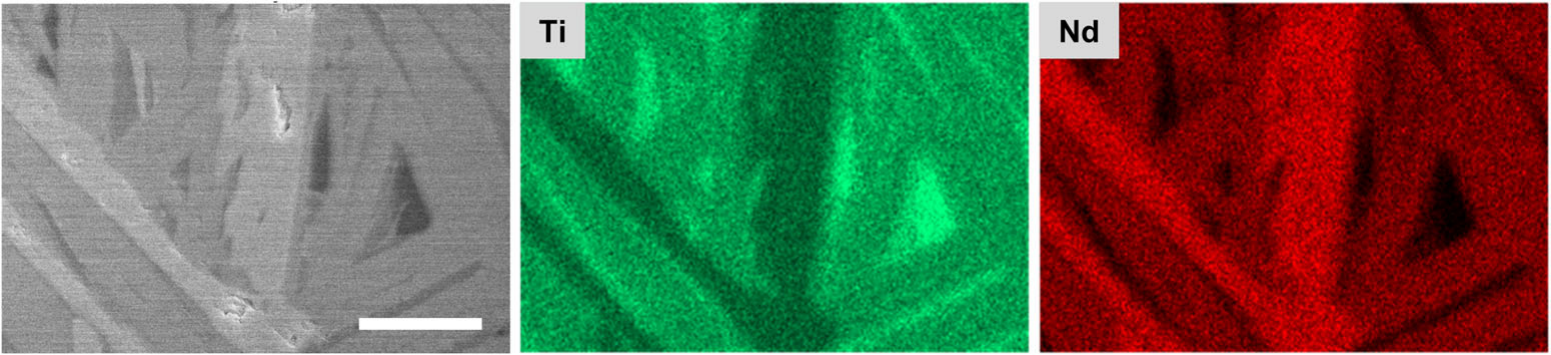
Energy dispersive spectroscopy maps of NT crystalline sample formed during melt processing in microgravity. The electron micrograph shows three contrasting regions of increasing brightness, corresponding most closely to previously identified phases TiO_2_, Nd_4_Ti_9_O_24_, and Nd_2_Ti_2_O_7_. The (fourth) darkest regions are voids in the sample. Scale bar is 10 μm.

Crystalline samples from melt quenching exhibited much more microstructural diversity between microgravity and terrestrial conditions. Crystalline terrestrial samples appeared homogeneous in X-ray tomography (Fig. 6e), except for small pores and cracks. The lack of density contrast suggests that the sample is single-phase or, if multi-phase, that its crystalline domains are smaller than ~8 μm (i.e., ~6 voxels wide, given the voxel size of (1.37 μm)^3^). The crystalline microgravity sample exhibited clear density contrast in tomography (Fig. 6b), with lamellar crystalline grains 4–10 μm thick and as wide as ~800 μm. Voids are present in the sample both in spherical form and as small gaps or cracks between crystalline domains. SEM (Fig. 7b) and energy dispersive spectroscopy (EDS, Fig. 8) reveal domains of three different compositions: 30.4(7), 17.3(2), and 2.5(1.3) mol. % Nd_2_O_3_. Of the previously identified crystalline phases for TiO_2_-Nd_2_O_3_^48^, these compositions correspond most closely to Nd_2_Ti_2_O_7_ (33.3 mol. % Nd_2_O_3_), Nd_4_Ti_9_O_24_ (18.2 mol. % Nd_2_O_3_), and TiO_2_.

**Fig. 9:**
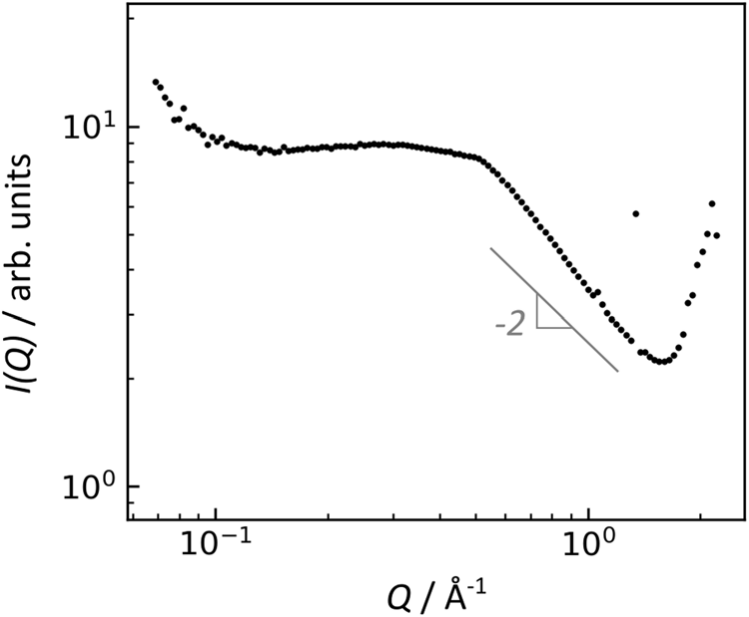
Small-angle X-ray scattering of NT glass-ceramic formed during melt processing in microgravity. The linear regime at *Q* = 0.6-1.0 Å^-1^ corresponds to the interfaces between crystalline and glassy domains.

The glass-ceramic prepared in microgravity also appeared homogenous in X-ray tomography (Fig. 6c) and SEM (Fig. 7c). Its crystalline phase is a minor component of the sample, based on the very weak Bragg peaks observed in its X-ray diffraction. Indexing the Bragg peaks does not provide a clear match to any of the expected phases (Supplementary Fig. 4). The closest match is Nd_2_Ti_2_O_7_, though some peak positions are shifted or missing, which may be due to a highly distorted crystal lattice and/or this measurement’s lack of orientational averaging. The glass-ceramic Bragg peaks may also match Nd_2_Ti_3_O_9_ with a considerable contraction of the lattice. Small-angle X-ray scattering (SAXS, Fig. 9) shows a linear Porod regime in the range of *Q* = 0.6–1.0 Å^−1^, which corresponds to the interfaces between crystalline and glassy domains. The Porod slope is close to the value of 2 expected for flat disc-shaped particles, which is consistent with the lamellar morphology of Nd_2_Ti_2_O_7_ crystal grains observed in the fully crystalline product (Fig. 6b). The SAXS exhibits a broad peak approximately centered at *Q* = 0.5 Å^−1^, corresponding qualitatively to a feature 2π/*Q* = 1.25 nm in size. The origin of this broad peak is unclear; it is too small for the crystallite size, given the Nd_2_Ti_2_O_7_ monoclinic unit cell^49^ with *a* = 7.67 Å, *b* = 13.00 Å, and *c* = 5.46 Å.

## Discussion

The discussion focuses on how melt processing in microgravity affects the solidification behavior, both for vitrification and crystallization. We begin with vitrification.

Fragile liquids generally have low glass-forming ability, as is the case for rare-earth titanates like NT^25^. Glasses of NT have been prepared by only two methods: roller quenching and containerless processing. In roller quenching, exceptionally high cooling rates (~10^6^ °C s^−1^) are the key to avoiding crystallization, and only thin flakes (tens of μm thick) of product can be obtained. In containerless processes such as levitation, the lack of surface contact eliminates heterogeneous nucleation of crystals in the sample, so glasses can be formed at much lower cooling rates (10^2^ to 10^3^ °C s^−1^) and with larger sample dimensions (spheres ~3 mm in diameter). These benefits make levitation a useful tool for glass research on fragile liquids, providing large enough samples to investigate application-specific properties and guide the development of new glasses.

For aerodynamic levitation, however, the sample size that can be vitrified is limited by fluid properties and/or heat transfer. Stable levitation requires that the liquid sample maintains a nearly spherical shape, so materials with low surface tension are challenging to process because liquid beads deform or fragment in response to the forces of gravity and gas flow around the sample. For fragile liquids with sufficient surface tension, the maximum sample size for glass formation is limited by the critical cooling rate for vitrification. As already discussed in Fig. 1b, the cooling rate (−*dT*⁄*dt*) is predominantly determined by radiative heat transfer at high temperatures (e.g., above 900 °C) and by convection at lower temperatures. As sample size increases, the smaller ratio of surface area to volume results in a smaller radiative cooling rate. Once the sample is sufficiently large, it can no longer cool faster than the critical cooling rate, so it will crystallize. In the terrestrial aerodynamic levitator, NT melts up to 53 mg consistently vitrified, while melts 66 mg or larger consistently crystallized during cooling (Supplementary Fig. 1e, f). Crystallization occurred reproducibly ca. 1040 °C, which is consistent with the sample that crystallized in microgravity (discussed further below). Based on comparison of samples with different masses, the critical cooling rate in the aerodynamic levitator is ~170 °C s^−1^ at 1040 °C.

The cooling rate in microgravity was consistently 40–110 °C s^−1^ lower than on Earth for NT samples of the same size (20–23 mg, Fig. 1b). This difference is mainly due to the forced convective cooling associated with aerodynamic levitation, which is absent in electrostatic levitation in microgravity. Because aerodynamic levitation is not well suited for the microgravity environment, these differences in cooling rate may also represent an inherent difference in melt processing for Earth vs. reduced gravity conditions. If the critical cooling rate is the same in both environments, larger samples may be more difficult to vitrify in microgravity.

The cooling rates for a microgravity sample (21 mg) and the largest terrestrial glass (53 mg) provide an interesting comparison. For this pair, near 1040 °C is a crossover point (Supplementary Fig. 1f), above which the microgravity cooling rate is faster and below which the microgravity cooling rate is slower, compared to the terrestrial 53 mg sample. Yet, the microgravity sample does not crystallize before reaching the glass transition at *T*_g_ = 786 °C, suggesting that the most crucial temperature range for investigating critical cooling rates is near or above the 1040 °C crossover temperature.

The atomic structures of glasses were nearly identical for the Earth and microgravity processing conditions, except for subtle differences that could be explained by compositional variations of ~2 mol. % Nd_2_O_3_. This comparison provides validation, at least for rare-earth titanates, that the same glass can be manufactured in space as on Earth, aside from differences in thermal history.

Next, we turn the discussion toward observations of crystallization in microgravity. One NT sample crystallized in microgravity and formed a highly unusual microstructure compared to the terrestrial crystalline samples (cf. Fig. 6b, e). The microgravity sample exhibits lamellar crystal grains of Nd_2_Ti_2_O_7_ (the brightest regions in Figs. 6b and 7b), with some individual lamellae spanning most of the entire sample. Many of these lamellae originate from a common internal point (Fig. 6b, red arrow; Supplementary Movie 6), suggesting that nucleation first occurred at this location.

The unusual crystal nucleation of this sample is likely due to a combination of unstable levitation and localized laser beam heating of the supercooled melt. When this sample in ELF was being held isothermally ca. 1750 °C, it began to drift in and out of the central levitation position where the four heating lasers were co-focused. (Levitation instability often occurs when the surface charge on the sample diminishes or changes polarity^50^.) The sample exited the lasers’ focal spot for ~1.5 s, then partially reentered the levitation position, and assumed a stable position such that the lasers’ focus was off-center on the sample. Then, sudden crystallization occurred, evidenced by the sample surface becoming faceted and loss of stable surface charge. From these observations and the clear nucleation point observed with tomography, it seems likely that the small laser focal spot (0.5 mm diameter) resulted in very localized heating, which led to crystal nucleation in the hot spot. This scenario is very different than the terrestrial aerodynamic levitator, in which a single, less focused laser provides sample heating.

The microgravity crystalline sample contained a mixture of three crystal phases, which can be explained using the equilibrium phase diagram^48^ (Supplementary Fig. 5), though not with certainty since the local temperature during nucleation is not known because the sample position was not aligned with the pyrometer. First, we assume the liquid is likely ~19 mol. % Nd_2_O_3_, based on X-ray diffraction and Raman spectroscopy. From tomography, we infer that Nd_2_Ti_2_O_7_ was the first phase to nucleate from this liquid, so the sample temperature was either 1500–1589 °C (between the solidus and liquidus) or below 1199 °C. (Between 1199 and 1500 °C, Nd_2_Ti_3_O_9_ is expected instead of Nd_2_Ti_2_O_7_.) The higher temperature range is most likely, given that the pyrometer corrected temperature was 1410 °C at the time of nucleation, and the local temperature at the lasers’ focal spot would be higher. As the Nd_2_Ti_2_O_7_ crystals grew, the melt would then become depleted in Nd_2_O_3_ until approaching the eutectic composition at 15.6 mol. % Nd_2_O_3_. Further cooling would then result in the crystallization of Nd_4_Ti_9_O_24_ and TiO_2_, consistent with the final mixture of phases observed in SEM/EDS.

## Methods

### Sample preparation and melt processing

Samples of nominal composition 83TiO_2_-17Nd_2_O_3_ (NT) were prepared from powders of TiO_2_ (99.98%, rutile, Aldrich) and Nd_2_O_3_ (99.999%, Cerac). Powders were dried in Pt crucibles at 600 °C for at least 3 h, then weighed, and mechanically mixed. Portions of the powder mixture were then heated and briefly melted in a copper hearth using a 10.6 μm CO_2_ laser. The resulting spheroids were then processed in an aerodynamic levitator^27^ using air as the gas stream, and they were remelted with laser beam heating. The melts were heated to 2000 °C for approximately 30 seconds and then freely cooled by turning off the laser. Samples for both the terrestrial and microgravity experiments were prepared following this procedure, although from different batches of powder mixtures due to an insufficient amount of the original mixture. This contributed to the compositional variations described in the Results section.

Cooling curves in the aerodynamic levitator were collected for samples of varying mass, 20–66 mg each, using pyrometers with λ = 0.9, 1.5, or 5.0 μm (Chino Corporation, spot size 0.6 mm diameter). Multiple cooling traces for each sample and condition were recorded at 100 Hz to ensure reproducibility. The apparent temperature was corrected assuming a sample emissivity of 0.86.

For microgravity experiments, samples were loaded to a sample cartridge for the ELF^16^ and launched to the International Space Station. Each sample was levitated and then laser beam heated. Levitation stability worsened upon heating as the sample charge decreased and switched polarity, particularly in the 1000–1400 °C range. If the sample motion could be controlled, upon melting the levitation would again become stable once thermionic emission created a stable surface charge. For the NT composition, 5 out of 6 heating attempts succeeded in melting the samples. The melts were heated to ~1850 °C and held isothermally for at least several minutes. Then, the lasers were turned off, and the sample cooled freely. Pyrometry data (IMPAC IGA140, λ = 1.55 μm, spot size 0.3–0.5 mm diameter) were collected at 100 Hz, and the apparent temperature was corrected assuming an effective emissivity of 0.63. This effective emissivity accounts for the multiplicative effects of two windows positioned between the pyrometer and the sample, and a sample emissivity of 0.86. The temperature uncertainty is ca. ±30 °C at 1800 °C and smaller at lower temperatures. A full discussion of the temperature correction estimations and temperature uncertainties is provided in the Supplementary Discussion.

Sample cooling rates were calculated from the pyrometry data. For each time point, a range of data ±0.5 s was defined and then fitted using a second-order polynomial. The instantaneous cooling rate was then calculated as the slope of the fitted polynomial, evaluated at the time point. This procedure was tested using different fitting ranges (±0.1, 0.5, or 1.0 s), and ±0.5 s was found to best fit the cooling data. However, subtle features like small recalescence events were not always resolved with this method, so cooling traces were manually assessed for recalescence.

### Density measurements and analysis

Samples’ densities were determined using their masses after melt processing and volume measurements made in ELF, as described previously^17,51^. As a brief summary, during melt processing a camera recorded backlit silhouette video of the sample (30 Hz, interlaced). Individual frames from these videos were extracted and deinterlaced, and an edge detection algorithm was used to define the perimeter of the sample. The sample volume was then calculated from the edge fitting using a Legendre polynomial series^18^. Volume calibration (mm^3^ pixel^−3^) was performed by analyzing video of stainless steel calibration spheres that were levitated in ELF at the beginning and end of each sample holders’ experiments. Lastly, the sample volumes were corrected for the internal porosity as determined from X-ray tomography.

Pyrometry data, collected at 100 Hz, were spline interpolated to a 60 Hz basis to align with the deinterlaced video frames. Sample volumes at a given temperature were compared for dynamic (i.e., during sample cooling) and isothermal measurements, which were found to be consistent.

### Atomic structure measurements of glasses

High-energy X-ray diffraction measurements were performed at Section 6-ID-D of the Advanced Photon Source, Argonne National Laboratory (Argonne, IL, USA). The diffracted intensity of 99.9 keV X-rays from whole samples was measured in transmission geometry using a two-dimensional area detector (Varex 4343CT) at a distance of ~329 mm from the sample. Samples were mounted on polyimide tape, and the sample was positioned so that the incoming X-ray beam (0.5 mm wide and 0.5 mm tall) passed through near the edge of the glass bead, so that X-ray transmission was 84–91%. The diffracted intensity was azimuthally integrated using Fit2D^52^, and the data were reduced and normalized in GudrunX^53^ to obtain the X-ray total structure factors, *S*(*Q*), following procedures described previously^26^. Free atom X-ray form factors were used according to Waasmaier and Kirfel^54^, and the “top hat” convolution in GudrunX^55^ was not applied. A sine Fourier transform was applied to the structure factors to obtain the differential PDFs, *D*(*r*):

5 $$D\left(r\right)=\frac{2}{\pi }{\int }_{0}^{{Q}_{\max }}Q\left(S\left(Q\right)-1\right)M(Q)\sin \left({Qr}\right){dQ}$$

where *ρ* = 0.08163 atoms Å^−3^ is the atomic number density, *M*(*Q*) is the Lorch modification function^56^, and *Q*_max_ = 21.8 Å^−3^.

To obtain mean bond distances and coordination numbers, NXFit^57^ was used to fit the first two PDF peaks using Gaussian distributions convolved with their associated peak-shape functions. For peak fitting, the total PDF was used, *T*(*r*) = *D*(*r*) + 4*πρr*. In NXFit, three Gaussian distributions were initially defined, one each for Ti-O, Nd-O, and O-O. The initial coordination numbers and O-O bond distances were taken from the published structural model^26^, and bond distances were set for Ti-O and Nd-O based on the X-ray PDF maximum peak positions. The peak fitting parameters were then refined over the range *r* = 1.335–2.60 Å using limits of ±0.04 Å for bond distances and ±0.8 for coordination numbers.

Neutron diffraction measurements were collected at the NOMAD beamline^58^ of the Spallation Neutron Source, Oak Ridge National Laboratory (Oak Ridge, TN, USA). Individual glass beads were loaded into 3 mm diameter thin-walled silica capillaries and measured. Because the scattering intensity from small samples is quite weak, measurements were also collected on each empty capillary so that the background subtraction would not leave silica signatures due to differences in the capillary wall thicknesses. At the sample position, the neutron beam was approximately Gaussian in shape with a FWHM of 6 mm. After background subtraction, the samples’ scattering intensities were normalized against that of a vanadium standard.

Raman spectroscopy was carried out on a WITec Alpha300 Raman Spectrometer, equipped with an 1800 grooves mm^−1^ diffraction grating with a ×50 objective lens. All measurements were made at room temperature with a resolution of 10 cm^−1^ or better using an excitation wavelength of 488 nm. Each spectrum is an average of 200 scans in the range of 85–1400 cm^−1^ accumulated in one-second intervals. Spectra were corrected for the Bose-Einstein thermal population factor and area normalized^59,60^. Peak fitting of the terrestrial glasses’ Raman spectra was carried out using commercial graphing software (OriginLab). Lineshapes of components were assumed to be pseudo-Voigt functions. The Lorentzian and Gaussian contributions were fixed at 60% and 40%, respectively^61–63^. The spectrum of TG2 glass (17.1 mol.% Nd_2_O_3_) was fit first, and the center frequencies of the bands were subsequently fixed while the bandwidths and areas were optimized. This methodology produced good fits for glasses with different Nd_2_O_3_ content, including TG1, though the constant center frequency constraint needed to be relaxed for a small shoulder band at ~828 cm^−1^. This feature, as discussed in the Results section, has a strong overlap with a high-intensity feature at ~740 cm^−1^. Good fitting results were obtained by allowing the position of this band to vary within the range 828 ± 5 cm^−1^.

### Microstructure measurements

X-ray microtomography measurements were performed at Sector 7-BM-B of the Advanced Photon Source. Incoming X-rays were attenuated with Cu and Ge filters to yield a polychromatic X-ray beam with a mean energy ca. 80 keV. Each sample was positioned in a parallel beam configuration, and X-ray transmission was detected with a 25 μm thick LuAG scintillator positioned in front of a ×5 objective lens and FLIR Oryx camera. The setup provided a pixel size of 1.37 μm in the recorded projections. For each tomography scan, 2001 projections were collected at equally spaced rotational increments between 0 to 180° inclusive with a camera exposure time of 20 ms, in addition to dark and background images. Tomographic reconstructions were obtained with TomoPy^64^ and TomoCuPy^65^, using the gridrec algorithm^66^ and accounting for beam hardening. Image analysis was performed in ImageJ, first for segmentation using an automatic threshold. Then, porosity was measured by recording the number of “solid” pixels before and after filling holes in the sample cross-section images.

For SEM and energy dispersive spectroscopy (SEM/EDS, Hitachi SU8030), samples were mounted in Crystalbond 509 (Buehler), mechanically grinded, and polished to a final step with 1 μm diamond suspension. At least ten EDS measurements were collected across four sites on each polished cross-section to assess compositional uniformity.

Small-angle X-ray scattering of the microgravity glass-ceramic sample was measured at Sector 9-ID-C of the Advanced Photon Source^67^. The sample was mounted with Scotch Magic Tape (3M), and scattering of 21 keV X-rays was measured in a transmission geometry. Data were reduced with Nika software^68^.

### Reporting summary

Further information on research design is available in the Nature Research Reporting Summary linked to this article.

### Supplementary information

Supplementary Information

Supplementary Movie 1. Three-dimensional tomographic reconstruction of a microgravity glass

Supplementary Movie 2. Three-dimensional tomographic reconstruction of the microgravity crystal

Supplementary Movie 3. Three-dimensional tomographic reconstruction of the microgravity glass-ceramic

Supplementary Movie 4. Three-dimensional tomographic reconstruction of a terrestrial glass

Supplementary Movie 5. Three-dimensional tomographic reconstruction of a terrestrial crystal

Supplementary Movie 6. Three-dimensional tomographic reconstruction of the microgravity crystal

Reporting Summary

## Data Availability

All data are available upon reasonable request.

## Code availability

All pertinent steps for analysis are detailed in the Methods section; no author-generated code is necessary. Code-related questions can be directed to the corresponding author.
