# Supplementary material for: Microgravity effects on nonequilibrium melt processing of neodymium titanate: thermophysical properties, atomic structure, glass formation and crystallization
Source: NPJ Microgravity. 2024 Mar 6;10:26. doi: 10.1038/s41526-024-00371-x (PMC10918169; doi:10.1038/s41526-024-00371-x)
Supplement: Supplementary file 1 — Supplementary Information [file 41526_2024_371_MOESM1_ESM.pdf]

Supplementary Information for:

Microgravity effects on nonequilibrium melt processing of neodymium titanate: thermophysical properties, atomic structure, glass formation and crystallization

Stephen K. Wilke, Abdulrahman Al-Rubkhi, Chihiro Koyama, Takehiko Ishikawa, Hirohisa Oda, Brian Topper, Elizabeth Tsekrekas, Doris Möncke, Oliver L. G. Alderman, Vrishank Menon, Jared Rafferty, Emma Clark, Alan L. Kastengren, Chris J. Benmore, Jan Ilavsky, Jörg Neuefeind, Shinji Kohara, Michael SanSoucie, Brandon Phillips, Richard Weber

SUPPLEMENTARY DISCUSSION

Pyrometry and temperature corrections

Temperature measurements in ELF used an optical pyrometer sensitive to 1.45-1.8 μm radiation, or nominally λ = 1.55 μm. The apparent temperature measured by the pyrometer, T_{app}, must be corrected to obtain the absolute temperature, T_{abs}, according to Wien's law:

$$\frac{1}{T_{abs}} - \frac{1}{T_{app}} = \frac{\lambda \ln(\epsilon_{eff})}{C_2} \tag{1}$$

In Supplementary Equation 1, λ is the pyrometer wavelength, C_2 = 1.4388 cm K is the second radiation constant, and ϵ_{eff} is the effective emissivity, which contains contributions from the sample and any windows (reflective surfaces) between the sample and pyrometer. In ELF, two sapphire windows separate the pyrometer's detector from the sample[1]. The index of refraction, n, and the reflection per window surface, R_0, are related by the Fresnel equation:

$$R_0 = \left|\frac{n_1 - n_2}{n_1 + n_2}\right|^2 \tag{2}$$

where n_1 = 1.77 for sapphire and n_2 = 1.0 for air, so R_0 = 0.0773. The sample emissivity is estimated as 0.86, based on the previously measured refractive index of 2.2 for lanthanum titanate glass[2]. This yields an effective emissivity of $\epsilon_{eff} = 0.86 \times (1 - 0.0773)^4 = 0.63$.

The effective emissivity was also estimated via a second approach, using the recalescence temperatures of materials with known melting points. During crystallization from a supercooled melt, the sample is expected to self-heat back up to its equilibrium melting point (T_m) as the latent heat of fusion is released. Using this assumption, the peak temperature of recalescence would correspond to T_m, from which ϵ_{eff} could be calculated using Supplementary Equation 1 and the measured T_{app}. However, this assumption can often fail for at least two reasons: (i) if the liquid has supercooled sufficiently, its latent heat is not sufficient to heat back to T_m (also called hypercooling); (ii) the solid's emissivity is often lower than that of the melt, due to loss of a specular, spherical surface. In both of these scenarios, ϵ_{eff} would be underestimated by this method based on the recalescence temperature. With that acknowledgement, several recalescence events were analyzed for materials other than the NT samples reported in this paper: Al_2O_3, Gd_2O_3, Tm_2O_3, lanthanum titanate, and barium titanates. Of this set, the ϵ_{eff} estimates typically ranged 0.6 to 0.7.

Based on these two approaches to estimating ϵ_{eff}, a value of 0.63 was used for all analyses in this study. The temperature uncertainty arising from $\epsilon_{eff} = 0.63 \pm 0.05$ is ± 30 °C at 1800 °C and is smaller at lower temperatures.

When liquids were held isothermally in ELF, pyrometer noise was typically ± 15 °C (standard deviation). Thus, the overall temperature uncertainty for the ELF measurements in this study is estimated as ± 30 °C.

In the terrestrial aerodynamic levitator, the temperature of liquid samples varies spatially by ~40 °C from top to bottom, since the single heating laser only heats the top of the sample.[3] Using the sample emissivity of 0.86 and appropriate window corrections, ϵ_{eff} = 0.79. The temperature measurement uncertainty arising from $\epsilon_{eff} = 0.79 \pm 0.05$ is ± 27 °C at 1800 °C and is smaller at lower temperatures.

Density analysis and possible measurement artifacts

Glass density at room temperature

A literature estimate for NT glass density is available from pycnometry measurements by Arai *et al.*[4] on lanthanum titanate (LT) glasses. In their study, LT with 18.2 mol. % La_2O_3 had a density of 4.89 g cm^{-3}, which is 98.6% of the density for the compositionally identical crystal phase $La_4Ti_9O_{24}$ (4.96 g cm^{-3}). Assuming the same ratio of glass-to-crystal density, NT glass with 18.2 mol. % Nd_2O_3 is expected to have a density of 5.10 g cm^{-3} (using $\rho = 5.18$ g cm^{-3} for crystalline $Nd_4Ti_9O_{24}$[5]). Arai *et al.* also observed a density increase of 2.3% in LT glasses as La_2O_3 content increased from 15.4 to 18.2 mol. %. Based on our atomic structural characterizations, the NT microgravity glasses likely contained ~19 mol. % Nd_2O_3, so a linear extrapolation of Arai's compositional trend would yield a final estimate of 5.13 g cm^{-3} for the MG1 and MG2 glasses here. Thus, the room temperature glass density according to the ELF measurements, 5.28 g cm^{-3}, is 2.9% larger than the literature-based estimate.

For comparison, the MG1 and MG2 glass densities at room temperature have been assessed using two other techniques in this study, X-ray tomography and light microscopy. The results are summarized in Supplementary Table 2 alongside the ELF measurement and literature-based estimate. The volume calculations from tomography and microscopy are assumed to have uncertainties of ca. 5%. In X-ray tomography, uncertainty arises from the image calibration factor and the thresholding algorithm used for image segmentation. For light microscopy, uncertainty arises from focal depth and assuming the sample is a perfect spherical shape. For ELF, an uncertainty of 2.5% is listed in Supplementary Table 2 based on prior reports[6]. For the estimate based on Arai *et al.*[4], no uncertainty is given in Supplementary Table 2 since no information on replicate measurements were reported. All four values for glass density in Supplementary Table 2 are almost within their collective uncertainties.

Anomalous thermal expansion near T_g

As discussed in the main text, the density-temperature relationship in Fig. 2 exhibits a steeper slope between 720-930 °C than either the glass or liquid regions. To explain this unexpected observation, several possible scenarios involving measurement artifacts were explored, including: (i) sample transparency to the silhouette backlight, (ii) changes in bubble volume inside the sample, and (iii) sample transparency at the pyrometer wavelength. These are discussed in detail below.

ELF measurements of liquid densities have been shown to be in good agreement with prior (terrestrial) studies, for example with Al_2O_3[1], Y_2O_3[7], lanthanoid sesquioxides[8], Ga_2O_3[9], Zr[10], and Au[11]. These examples provide validation of the ELF instrument for measuring liquid density. However, the measurements here for NT are the first to be reported from ELF for glass density upon cooling, so we explored whether transparency of the glassy state may be leading to an underestimation of the sample volume. Specifically, if the sample becomes partially transparent near the wavelength of the ultraviolet backlight, some of the backlight may pass through the edges of the sample, making its silhouette appear smaller in the camera image than the true size. Sample volume is calculated based on edge detection of this silhouette image (see Methods section). The typical ELF sample has a radius of ~120 pixels in the camera image, so if sample transparency introduces an error of ~1 pixel to the edge detection algorithm, a volume error of ~2.5% would result. This error may onset suddenly during cooling if the sample transparency changes at a particular temperature, which matches what is observed in the anomalous density increase (i.e., volume decrease) ca. 720-930 °C near $T_g = 786$ °C. For illustrative purposes, Supplementary Fig. 6a compares the measured data against the hypothetical "true" density suggested by this explanation (green lines). To test this hypothesis, precision spheres of optically-transparent ruby, sapphire, and BK7 glass were levitated at room temperature under vacuum in a ground-based electrostatic levitator that uses the same silhouette imaging technique. The measured volumes of all three standards matched the expected values within 0.5%. This provides strong evidence against the hypothesis of edge transparency causing an underestimation of sample volume.

A second hypothesis for explaining the anomalous 720-930 °C region is that gas bubbles inside the samples shrank suddenly near 930 °C and then became fixed in size as the sample became viscous near T_g. The shrinking bubbles would result in the apparent sample volume decreasing, and the calculated density would increase. Supplementary Fig. 6b provides a comparison of the measured data and the hypothetical density suggested by this explanation. Since the internal voids of the glass samples were measured to be ≤ 0.25% of the total volume, the gas bubbles would have to be 10× larger in the melt to account for the magnitude of the density discrepancy. This seems unlikely. Furthermore, the two replicate samples exhibited reproducible density-temperature relationships (Fig. 2), and it is unlikely that such a bubble process would occur identically in both samples.

A third hypothesis involves the sample becoming partially transparent at the pyrometer wavelength during cooling. If the transition to partial transparency began near 930 °C, then the pyrometer would start seeing some of the radiation emitted by the sample's hotter interior. This would result in a higher reading than the surface temperature, until the sample interior had also cooled enough to become partially transparent. Supplementary Fig. 6c shows a comparison of the measured data and the hypothetical density suggested by this explanation. This scenario seems the most convincing of the considered measurement artifacts, so it was presented in the main text.

SUPPLEMENTARY FIGURES

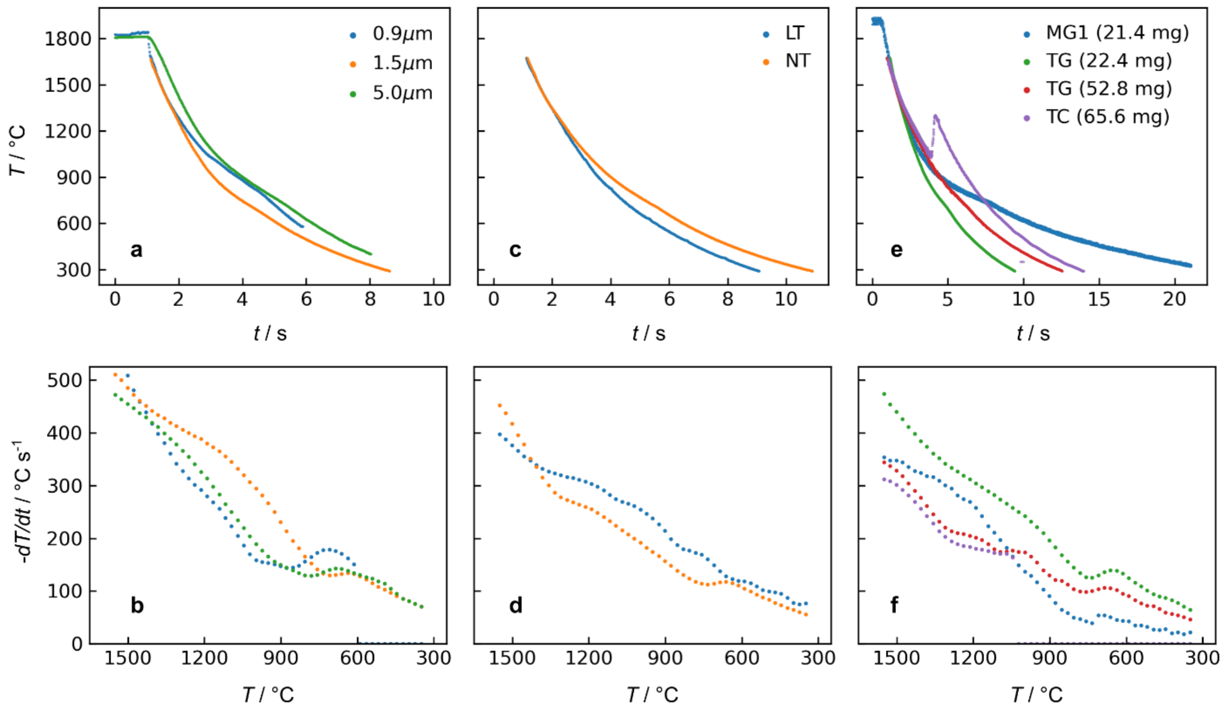

Supplementary Fig. 1. Pyrometry and cooling rates of molten titanates processed using the terrestrial aerodynamic levitator. a, Cooling curves and **b**, cooling rates for a single NT sample (21.8 mg) in pure O_2 gas measured with three different pyrometers: λ = 0.9, 1.5, or 5.0 μm. **c, d,** Comparison of rare earth titanates (nominally $83TiO_2$-$17RE_2O_3$) containing either RE = La ("LT", 33.8 mg total mass) or Nd ("NT", 32.6 mg). **e, f,** Comparison of one sample vitrified in microgravity (MG1) with three terrestrial samples of different masses. Terrestrial samples of 22.4 or 52.8 mg mass formed glass (TG), while a 65.6 mg sample crystallized (TC) upon supercooling to ~1040 °C. All data in **c-f** are for processing in air and collected at λ = 1.5 μm.

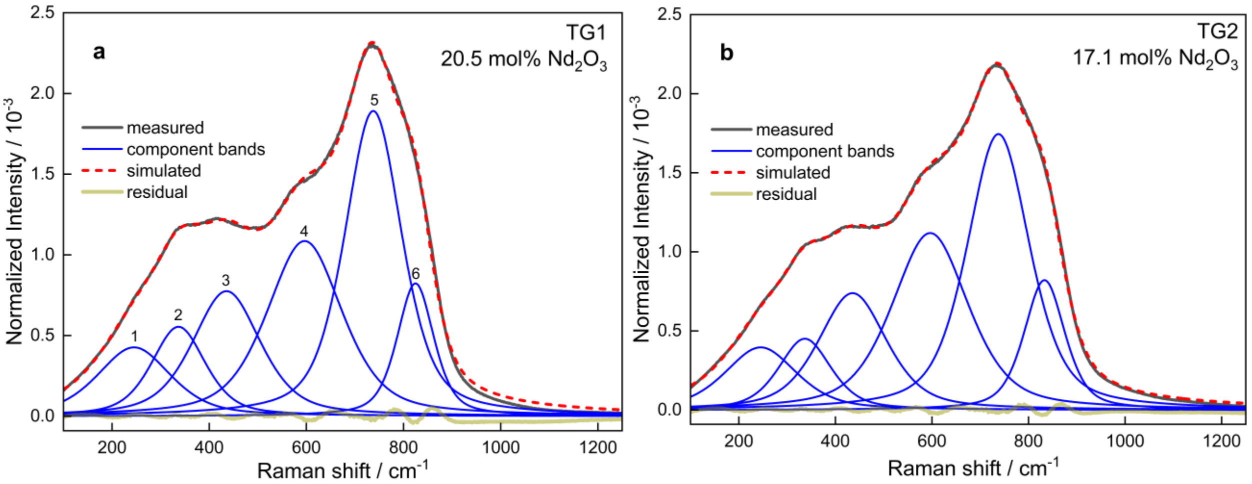

Supplementary Fig. 2. Fitting of component bands in Raman spectra. a, TG1 and **b,** TG2 terrestrial glasses. Optimized fitting parameters are listed in Supplementary Table 1.

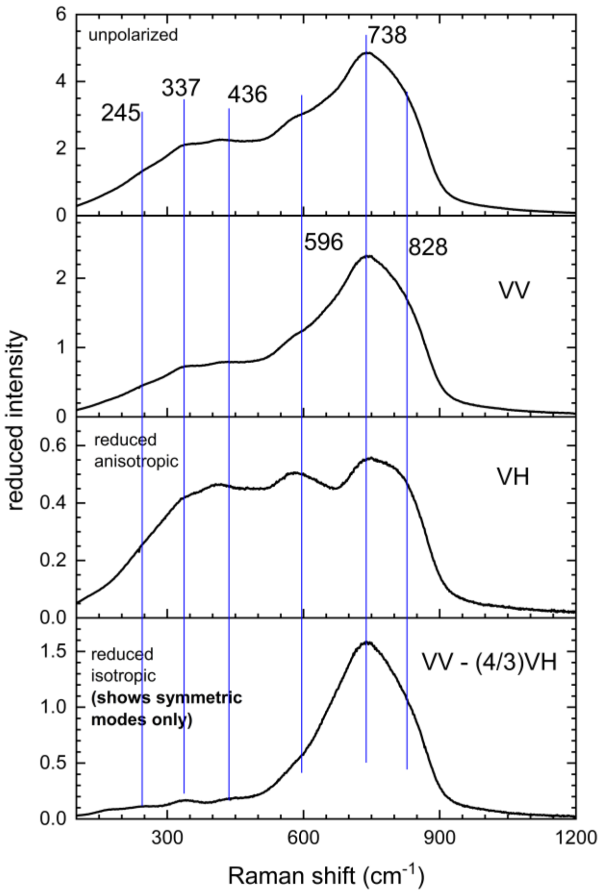

Supplementary Fig. 3. Comparison of unpolarized and polarized Raman spectra. For microgravity glass MG1. From top to bottom: unpolarized, parallel (VV) and perpendicular (VH) polarization, and the reduced isotropic spectrum, which shows only symmetric modes that are found in the high frequency envelope. Annotated bands are summarized in Supplementary Table 1 and discussed in the main text.

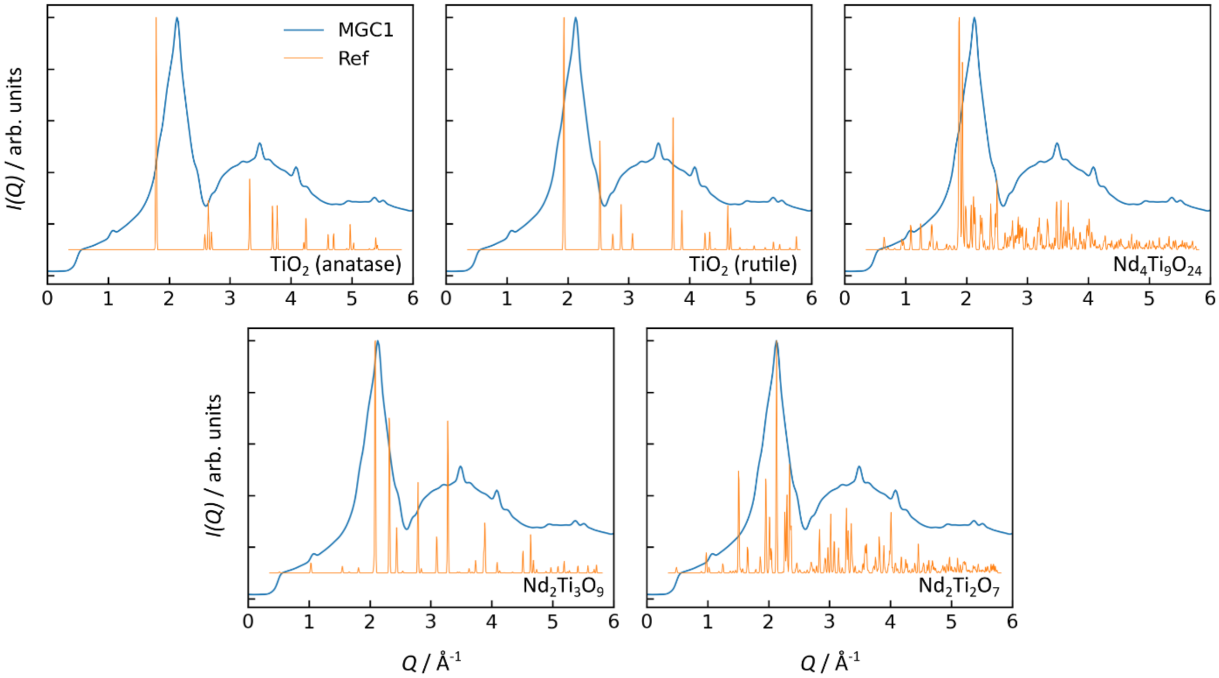

Supplementary Fig. 4. X-ray diffraction intensity for NT glass-ceramic processed in microgravity (MGC1). The small Bragg peaks in the MGC1 diffraction are compared with reference patterns for possible matching crystalline phases: TiO_2 (anatase)[12], TiO_2 (rutile)[13], $Nd_4Ti_9O_{24}$[5], $Nd_2Ti_3O_9$[14], and $Nd_2Ti_2O_7$[15].

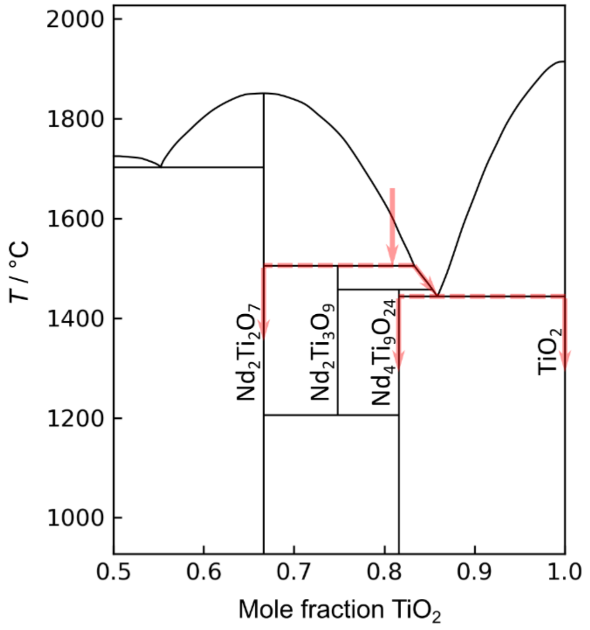

Supplementary Fig. 5. A portion of the Nd_2O_3-TiO_2 equilibrium phase diagram. Adapted from Gong and Zhang[16], showing likely solidification pathway (in light red shading) for the NT sample that crystallized during melt processing in microgravity. Annotations mark the line phases discussed in the main text.

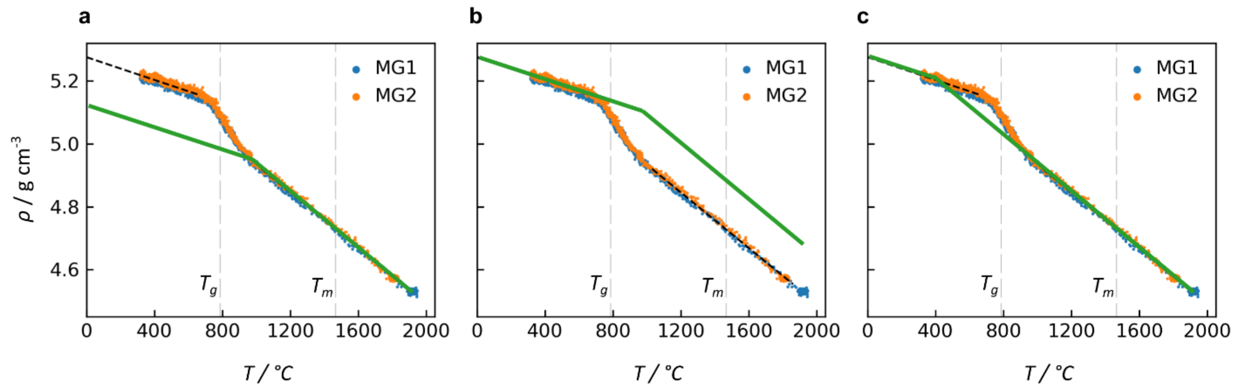

Supplementary Fig. 6. Hypothetical measurement artifacts to explain the anomalous density-temperature region ca. 720-930 °C. **a,** Sample transparency to the silhouette backlight, leading to underestimation of sample volume below 930 °C; **b,** large bubbles in the melt that shrink as the sample approaches T_g; **c,** sample transparency at the pyrometer wavelength. The green lines illustrate the hypothetical "true" density that is suggested by each of these scenarios.

SUPPLEMENTARY TABLES

Supplementary Table 1. Raman peak assignments for NT glasses. Peak fitting results are shown in Supplementary Fig. 2.

Position (cm^{-1})	Assignment	Reference	TG1 Height ($\times 10^2$)	Width	TG2 Height ($\times 10^2$)	Width
245	Network deformation and/or RE-O stretching	[17,18]	0.04264	179	0.03963	176
337	RE-O stretching and/or bending associated with corner-sharing TiO_6 units	[17,18]	0.0554	131	0.04511	128
436	Ti-O-Ti bending	[19,20]	0.07748	165	0.07392	167
596	Symmetric stretching of edge-sharing TiO_6	[19,20]	0.10854	184	0.11191	187
738	Symmetric stretching of corner-sharing TiO_6	[19,20]	0.18913	140	0.17447	153
~828	TiO_5	[19–24]	0.08227	89	0.08213	100

Supplementary Table 2. Estimates of NT glass density.

**Method**	**Density (g cm^{-3})**
ELF	5.28(13)
Tomography	4.86(24)
Light microscopy	5.20(26)
Pycnometry, Arai *et al.*[4]	5.13

SUPPLEMENTARY REFERENCES

1. Tamaru, H. *et al.* Status of the Electrostatic Levitation Furnace (ELF) in the ISS-KIBO. *Microgravity Sci. Technol.* **30**, 643–651 (2018).
2. Masuno, A. *et al.* Glass-forming region and high refractive index of TiO2-based glasses prepared by containerless processing. *Phys. Status Solidi C* **9**, 2424–2427 (2012).
3. McCormack, S. J., Tamalonis, A., Weber, R. J. K. & Kriven, W. M. Temperature gradients for thermophysical and thermochemical property measurements to 3000 °C for an aerodynamically levitated spheroid. *Rev. Sci. Instrum.* **90**, 15109 (2019).
4. Arai, Y., Itoh, K., Kohara, S. & Yu, J. Refractive index calculation using the structural properties of La4Ti9O24 glass. *J. Appl. Phys.* **103**, 094905 (2008).
5. Hübner, N. & Gruehn, R. Nd4Ti9O24: Präparation und Struktur. *Zeitschrift für Anorg. und Allg. Chemie* **616**, 86–94 (1992).
6. Ishikawa, T., Koyama, C., Oda, H. & Saruwatari, H. Status of the Electrostatic Levitation Furnace in the ISS - Surface Tension and Viscosity Measurements. *Int. J. Microgravity Sci. Appl.* **39**, 390101 (2022).
7. Oda, H., Shimonishi, R., Koyama, C., Ito, T. & Ishikawa, T. Determining the density of molten Y2O3 using an electrostatic levitation furnace in the International Space Station. *High Temp. - High Press.* **53**, 341–350 (2023).
8. Koyama, C. *et al.* Densities of liquid lanthanoid sesquioxides measured with the electrostatic levitation furnace in the ISS. *J. Am. Ceram. Soc.* **104**, 2913–2918 (2020).
9. Yoshida, K. *et al.* Thermophysical properties of molten Ga2O3 by using the electrostatic levitation furnace in the International Space Station. *Appl. Phys. Express* **15**, 085503 (2022).
10. Nawer, J. & Matson, D. M. Quantifying facility performance during thermophysical property measurement of liquid Zr using Electrostatic Levitation. *High Temp. - High Press.* **52**, 123–138 (2023).
11. Nawer, J. *et al.* Uncertainty analysis and performance evaluation of thermophysical property measurement of liquid Au in microgravity. *npj Microgravity* **9**, 38 (2023).
12. Horn, M., Schwerdtfeger, C. F. & Meagher, E. P. Refinement of the structure of anatase at several temperatures. *Zeitschrift für Krist.* **136**, 273–281 (1972).
13. The crystal structure of rutile as a function of temperature up to 1600°C. *Zeitschrift für Krist. - Cryst. Mater.* **194**, 305–313 (1991).
14. Richard, M., Brohan, L. & Tournoux, M. Synthesis, Characterization, and Acid Exchange of the Layered Perovskites: A2Nd2Ti3O10 (A - Na, K). *J. Solid State Chem.* **112**, 345–354 (1994).
15. Ishizawa, N., Ninomiya, K., Sakakura, T. & Wang, J. Redetermination of Nd2Ti2O7: a non-centrosymmetric structure with perovskite-type slabs. *Acta Crystallogr.* **E69**, i19 (2013).
16. Gong, W. & Zhang, R. Phase relationship in the TiO2-Nd2O3 pseudo-binary system. *J. Alloys Compd.* **548**, 216–221 (2013).
17. Topper, B., Tagiara, N. S., Herrmann, A., Kamitsos, E. I. & Möncke, D. Yttrium and rare-earth modified lithium orthoborates: Glass formation and vibrational activity. *J. Non. Cryst. Solids* **575**, 121152 (2022).
18. Sasaki, S. *et al.* Structural Origin of Additional Infrared Transparency and Enhanced Glass-Forming Ability in Rare-Earth-Rich Borate Glasses without B–O Networks. *Inorg. Chem.* **59**, 13942–13951 (2020).
19. Su, Y., Balmer, M. Lou & Bunker, B. C. Raman Spectroscopic Studies of Silicotitanates. *J. Phys. Chem. B* **104**, 8160–8169 (2000).
20. Möncke, D., Lind, F., Topper, B. & Kamitsos, E. I. Anomalous Deformation Behavior in ULE Glass upon Microindentation: A Vibrational Spectroscopic Investigation in the Induced Structural Changes of a Ti-Silicate Glass. *J. Phys. Chem. C* **125**, 4183–4195 (2021).
21. Henderson, G. S. & Fleet, M. E. The structure of Ti silicate glasses by micro-Raman spectroscopy. *Can. Mineral.* **33**, 399–408 (1995).

22. Osipov, A. A., Liška, M., Osipova, L. M., Chromčiková, M. & Hruška, B. Thermodynamic modeling and Raman spectroscopy study of Na2O-TiO2-SiO2 glasses. *Vib. Spectrosc.* **111**, 103160 (2020).
23. Richter, S. *et al.* Ultrashort pulse induced modifications in ULE - from nanograting formation to laser darkening. *Opt. Mater. Express* **5**, 1834–1850 (2015).
24. Efthimiopoulos, I. *et al.* Femtosecond laser-induced transformations in ultra-low expansion glass: Microstructure and local density variations by vibrational spectroscopy. *J. Appl. Phys.* **123**, 233105 (2018).
